# Acknowledgement to Reviewers of *Vaccines* in 2017

**DOI:** 10.3390/vaccines6010004

**Published:** 2018-01-09

**Authors:** Vaccines Editorial Office

**Affiliations:** MDPI AG, St. Alban-Anlage 66, 4052 Basel, Switzerland

Peer review is an essential part in the publication process, ensuring that *Vaccines* maintains high quality standards for its published papers. In 2017, a total of 51 papers were published in the journal. Thanks to the cooperation of our reviewers, the median time to first decision was 21 days and the median time to publication was 49 days. The editors would like to express their sincere gratitude to the following reviewers for their time and dedication in 2017:

Admon, ArieLoré, KarinAlbrecht, RandyLou, SongAlexander-Miller, Martha A.Lyon, GeorgeAllain, Jean-PierreMa, WenjunAlonso, CovadongaMarc, DanielAzab, WalidMatoba, NobuyukiBabiuk, ShawnMaynard, JenniferBachmann, MartinMcKenney, JennieBaliraine, FrederickMcVey, David ScottBánki, ZoltánMena, IgnacioBansal, Geetha P.Modhiran, NaphakBartee, EricMoe, Gregory R.Basaras, MirenMoiré, NathalieBinder, RobertMonji, AkiraBose, SayantanMonroy, FernandoBowden, ScottMonteiro, MarioBrun, AlejandroMoog, ChristianeBurns, JamesMoorman, Jonathan P.Busca, AureliaMusch, MarkCampbell, EdwardNachbagauer, RaffaelChen, QingxiaNagarajan, Uma M.Chen, KangNiederwerder, Megan C.Chopra, TeenaNisole, SebastienCoccia, ElianaNitsch-Osuch, A.Comas-Garcia, AndreuO’Connell, CatherineCookson, Susan T.Olivier, AliciaCorson, StephenOshiumi, HiroyukiDaftarian, PirouzParida, SatyaDawson, EricaPeckham, ErinDe Vries, RoryPeeples, MarkDel Real, GustavoPenmetcha, KumarDelhon, GustavoPentakota, Sri RamDiamond, Don J.Pentel, PaulEpstein, AlanPepin, MichelFerrini, SilvanoPerrier, JosetteFox, AnnetteRakasz, EvaFrahm, NicoleReid, St PatrickFrey, ChristianRestivo, VincenzoFriedland, RobertRidley, David B.Fukushi, HidetoRockman, StevenGaudreault, Natasha NRogerson, Stephen J.Geary, SeanRomano, MartaGehring, Adam J.Rota, Paul A.Gill, ChristopherRubino, SalvatoreGomez, GabrielaSamad, LamiyaGreber, UrsSampathi, ShilpaGreenberg, Richard N.Sarathy, Vanessa V.Griffiths, AnthonySautto, Giuseppe A.Groll, Andreas H.Schepens, BertHaber, PeninaSchotsaert, Michael A.Hattori, ToshioSharafeldin, TamerHe, QinshuiShibata, TakumaHefferon, Kathleen LauraShokal, UpasanaHoeben, RobSizun, ChristinaHolst, JohanSlifka, MarkInchley, Christopher S.Smith, AlysonIvanov, PavelSoema, Peter C.Jegaskanda, SinthujanSolinís, M.ÁngelesJohnson, Reed F.Spencer, Juliet V.Jones, JeremyStevens, Joanne M.Julkunen, IlkkaStewart, Cameron R.Kanellopoulos-Langevin, ColetteStrohfus, Pamela K.Kavanagh, OwenSuárez, Cinta PrietoKelly, BenSuzuki, YasuhiroKhan, Imtiaz A.Tang, QiyiKido, HiroshiTaylor, GeraldineKumar, MukeshTerrier, OlivierLafontaine, EricThomas, Stephen J.Landolt, Gabriele A.Twigg III, HomerLangridge, WilliamVan De Ven, RienekeLe, Hoang-ThanhVanpouille-Box, ClaireLe Moigne, VincentWatson, Alan M.Le Nouen, CyrilWilusz, JeffLea, SuzanneYamanaka, TakashiLee, SujinZakhartchouk, AlexanderLeitão, Jorge Humberto GomesZeng, WeiguangLeitner, GabrielZhang, HongLokhandwala, ShehnazZhang, DuoLópez, Carolina B.Zhu, Wandi

